# Analysis of NEC cases registered in the human milk bank database

**DOI:** 10.3389/fped.2025.1679676

**Published:** 2025-09-17

**Authors:** Katsumi Mizuno, Yuka S. Wada, Motoichiro Sakurai, Yuki Tani, Masafumi Miyata, Jun Shindo, Shigeru Nishimaki, Hiroki Den

**Affiliations:** ^1^ Department of Pediatrics, Showa Medical University, Tokyo, Japan; ^2^ Division of Neonatology, Center for Maternal-Fetal, Neonatal, and Reproductive Medicine, National Center for Child Health and Development, Tokyo, Japan; ^3^ Department of Pediatrics and Neonatology, Kameda Medical Center, Chiba, Japan; ^4^ Department of Pediatrics, Nara Medical University, Nara, Japan; ^5^ Department of Pediatrics, Fujita Health University School of Medicine, Aichi, Japan; ^6^ Department of Neonatology, Tokyo Metropolitan Children’s Medical Center, Tokyo, Japan; ^7^ Department of Pediatrics, Yokohama City University, Yokohama, Japan; ^8^ Department of Hygiene, Public Health, and Preventative Medicine, Showa Medical University, Tokyo, Japan

**Keywords:** donor human milk, necrotizing enterocolitis, human milk bank, preterm infants, fortifier, ELBWI, hemodynamic instability, PDA

## Abstract

**Background:**

Necrotizing enterocolitis (NEC) remains a major cause of morbidity and mortality in extremely low birth weight infants (ELBWIs), despite widespread donor human milk (DHM) use. This study examined NEC cases among DHM recipients to explore potential contributing factors.

**Methods:**

We retrospectively analyzed 1,425 infants registered in Japan's human milk bank database (2018–2023). NEC cases at Bell stage ≥ II were confirmed by attending physicians. Infants who received DHM only after NEC onset were excluded. Cases were categorized by onset timing and associated clinical factors.

**Results:**

Among 1,324 very low birth weight infants, 21 (1.58%) developed NEC, with 20 requiring surgical intervention. Median gestational age and birth weight were 25.1 weeks and 637 g, respectively. NEC onset was classified as follows: within 7 days of birth (*n* = 5), after 2 months (*n* = 5), after formula or fortifier use (*n* = 6), associated with hemodynamic changes (*n* = 7), or of unknown etiology (*n* = 4). Common factors included symptomatic PDA, congenital heart disease, infection, formula exposure, and ophthalmologic procedures.

**Conclusion:**

NEC can develop despite DHM use, often in association with early infections, PDA, or fortification. Strategies to further reduce NEC incidence should include management of hemodynamic instability, delayed formula introduction, and use of exclusive human milk-based diets. Further research should explore potential roles of ophthalmologic interventions and human milk fortifiers in NEC development.

## Introduction

Even in the 2015 national survey, the in-hospital mortality rate of extremely low birth weight infants (ELBWIs) in NICUs remained below 10%, demonstrating that Japan's neonatal care is among the best in the world. However, the proportion of necrotizing enterocolitis (NEC) and focal intestinal perforation as causes of death has shown an increasing trend—from 7.2% in 2005 to 14.1% in 2010, and 16.2% in 2015. The mortality rate for infants diagnosed with NEC was reported to be 39.6% (1). Additionally, approximately 40% of NEC survivors develop neurodevelopmental impairment (NDI), with significantly higher incidence in the NEC group compared to those without NEC (2). Therefore, preventive strategies against NEC are essential to further improve the prognosis of ELBWIs.

Various strategies have been introduced to reduce NEC, with some reports even documenting complete elimination of NEC cases (3). These strategies include establishing human milk banks, standardizing enteral nutrition, using probiotics, and oropharyngeal administration of colostrum (4). In Japan, NICUs have long encouraged the use of mothers' own milk, and both probiotics and oropharyngeal colostrum application are already widely practiced (5, 6), which may contribute to the country's relatively low NEC incidence.

Breast milk contains immunologically active substances such as secretory IgA, macrophages, lactoferrin, human milk oligosaccharides (HMOs), and exosomes, which offer superior NEC prevention compared to formula milk. Although donor human milk (DHM) undergoes pasteurization—reducing levels of secretory IgA and lactoferrin—studies indicate that HMOs and exosomes retain both their quantity and activity (7, 8). In California, the increased use of DHM in hospitals (from 27 hospitals in 2007 to 55 in 2013) was associated with a decrease in NEC incidence from 6.6% to 4.3% (9).

Standardization of enteral nutrition has also been shown in systematic reviews to significantly reduce NEC risk (risk ratio 0.22; 95% confidence interval 0.13–0.36) (10). However, this is not feasible when relying solely on mothers' own milk, highlighting the need for a human milk banking system. By incorporating human milk banks and standardized enteral nutrition into existing practices such as oropharyngeal colostrum administration and probiotic use, further reductions in NEC incidence in Japan are anticipated. In Japan, DHM is processed using Holder pasteurization. Milk from three donor mothers is pooled per batch. DHM is distributed within 6 months from the date of expression. This study analyzed NEC cases using a database of infants who received DHM to explore clinical patterns and contributing factors that may inform future prevention strategies.

## Materials and methods

In Japan, all infants who received DHM are registered in the human milk bank database. Parental consent has been obtained for inclusion in the human milk bank registration system, and this consent includes permission for the data to be analyzed and published in scientific research. The human milk bank registry captures infants who received donor human milk (DHM); infants who did not receive DHM (including those exclusively fed mother's own milk) are not included in this database. We analyzed data from 2018 to 2023. Infants who began receiving DHM after developing NEC were excluded from this analysis, even though they were included in the database. Due to the limited number of NEC cases (*n* = 21), only descriptive statistics were used. Continuous variables are presented as medians and interquartile ranges, and categorical variables as counts and percentages. No inferential statistical tests were performed, as the small sample size precluded meaningful quantitative analysis.

The following categories were used in this study:

• Birth weight and gestational age
• Postnatal age (hours/days) at initiation of enteral feeding with DHM, mother's own milk (MOM), and formula
• NEC diagnosis at Bell stage II or higher (Yes/No)
• For NEC cases, the postnatal day of diagnosis and associated clinical course

NEC cases were categorized by onset circumstances:

1. Onset within 7 days of birth
2. Onset after approximately 2 months
3. Onset following formula feeding or fortification with cow's milk-based fortifiers
4. Cases associated with hemodynamic changes
5. Cases without a clear associated factor

These categories were selected to reflect the timing and context of NEC onset, aiming to explore recurring clinical patterns rather than infer causality.

## Results

**Figure 1:**
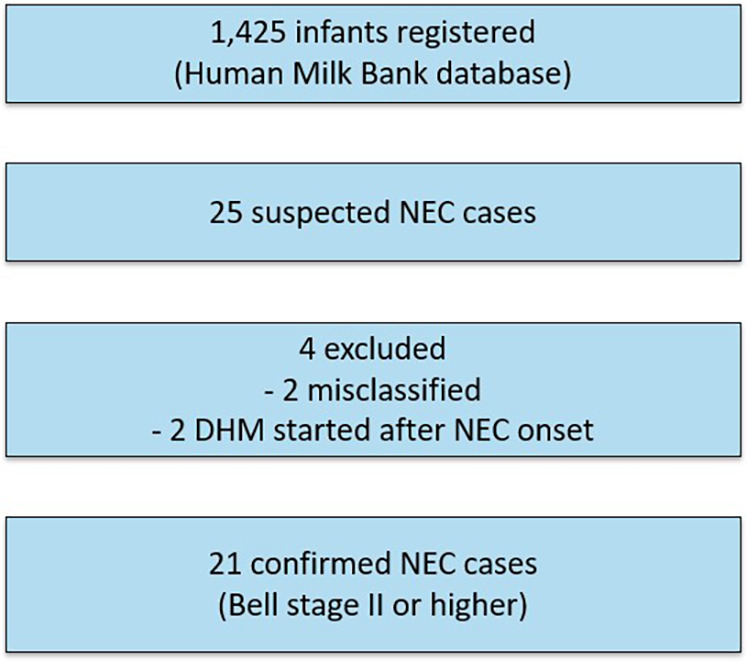
Flowchart of case selection. A total of 1,425 infants were registered in the Human Milk Bank database. Among these, 25 were reported as suspected NEC cases. Four cases were excluded (two were misclassified and two had donor human milk introduced only after NEC onset). The final study cohort therefore consisted of 21 confirmed cases of NEC at Bell stage II or higher.

A total of 1,425 infants (690 females, 735 males) were registered in the human milk bank database as of December 31, 2023. This included 1,324 very low birth weight infants, of whom 774 were classified as ELBWIs. Among these, 25 cases (1.75%) were recorded as NEC. A flowchart of case selection and exclusions is shown in Figure 1. Baseline clinical characteristics of the NEC cases are summarized by onset category in Table 1. To comply with de-identification requirements, gestational age (GA) and birth weight (BW) in Table 1 are presented in rounded values (completed weeks for GA and the nearest 100 g for BW). However, the median and interquartile range (IQR) values for each subgroup were calculated from the original data and are therefore shown with exact values.Consistent with the severity distribution (Bell stage IIb–III), nearly all infants required surgical intervention. NEC onset details were verified via email communication with attending physicians. Two cases were later determined not to have developed NEC, and two received DHM after NEC onset. Thus, 21 cases (1.58%; 19/1,324) were confirmed as Bell stage II or higher NEC, with 20 requiring surgical intervention. The total volume of DHM indicates that while the majority of infants received relatively small volumes of DHM (under 100 ml), a subset required over 1,000 ml, and in three cases, more than 5,000 ml was administered.

**Table 1:** Clinical characteristics of NEC cases by onset category.

Category	Case	GA (weeks)	BW (g)	Sex	First EN (h)	CS	Total DHM Volume(ml)	First FM (DoA)	NEC Onset (DoA)	NEC Stage	Surgery	Outcome	Complications	Symptoms by Fortifier
Early-onset	1	25	600	M	11	+	<50	158	3	III	+	Alive	CLD, ROP	−
Early-onset	2	25	700	M	9	+	<50	NA	4	III	+	Deceased	Severe infection	NA
Early-onset	3	26	1,000	M	11	-	50–99	31	4	III	+	Alive	sPDA, CLD	Abdominal distention
Early-onset	4	26	900	M	24	-	2,500–5,000	39	6	III	+	Alive	sPDA, ROP	−
Early-onset	5	24	700	M	27	-	50–99	28	7	III	+	Alive	sPDA, CLD, ROP	−
Median (IQR)		25.1 (24.7–26.7)	749 (650–988)		11 (9–26)	2/5					5/5			
Late-onset	6	23	600	M	5	+	<50	NA	56	III	+	Alive	CLD, ROP, IVH	−
Late-onset	7	37	1,300	F	69	-	<50	129	71	II	-	Alive	CoA	−
Late-onset	8	23	600	F	24	+	<50	NA	75	III	+	Alive	CLD, ROP	−
Late-onset	9	28	500	M	18	+	>5,000	15	131	III	+	Alive	Hernia	Abdominal distention
Late-onset	10	25	500	M	9	+	100–499	18	150	III	+	Alive	Hernia, CLD, ROP	−
Median (IQR)		25.3 (23.6–37.3)	594 (466–1,341)		18 (9–69)	4/5					4/5			
Formula/Fortifier	11	25	500	M	8	-	1,000–1,999	12	29	III	+	Alive	-	−
Formula/Fortifier	12	24	400	F	8	+	<50	13	38	III	+	Alive	CLD	−
Formula/Fortifier	13	29	600	M	13	+	<50	37	14	III	+	Alive	CLD, ROP	−
Formula/Fortifier	14	26	800	M	10	+	100–499	69	32	III	+	Alive	CLD, ROP	Abdominal distention
Median (IQR)		26.0 (24.3–29.0)	571 (433–762)		9 (8–13)	3/4					4/4			
Hemodynamic	15	23	600	M	28	+	50–99	9	10	III	+	Alive	CLD, ROP	−
Hemodynamic	16	27	1,000	M	72	+	>5,000	48	10	III	+	Alive	PDA clipping (Day 5)	−
Hemodynamic	17	28	400	M	9	+	50–99	21	16	III	+	Deceased	AV block	−
Median (IQR)		27.4 (23.6–28.7)	600 (433–996)		28 (9–72)	3/3					3/3			
Unknown	18	23	700	M	12	+	50–99	NA	20	III	+	Deceased	-	−
Unknown	19	23	400	M	12	-	100–499	34	23	III	+	Alive	CLD, ROP	−
Unknown	20	24	700	M	25	+	<50	74	24	III	+	Alive	ROP	Abdominal distention
Unknown	21	24	400	F	8	+	<50	13	32	III	+	Alive	CLD	−
Median (IQR)		24.1 (23.4–24.7)	542 (373–661)		12 (8–25)	3/4					4/4			

GA and BW are rounded for de-identification; subgroup medians (IQR) are calculated from original data.

GA, gestational age; BW, birth weight; M, male, F, female, EN, enteral nutrition; CS, colostrum, FM, formula milk; DoA, day of age; DHM, donor human milk; PDA, patent ductus arteriosus; CLD, chronic lung disease; ROP, retinopathy of prematurity; IVH, intraventricular hemorrhage; CoA, coarctation of the aorta.

Values are presented as median (IQR). Categories are defined based on the timing and clinical context of NEC onset.

The overall median gestational age and birth weight of these 21 NEC cases were 25.1 weeks (IQR 23.9–27.0 weeks) and 637 g (IQR 484.5–755.5 g), respectively.

The NEC cases were categorized (with some overlaps) as follows (Table 1):

1. Onset within 7 days of birth (5 cases)
2. Onset after approximately 2 months (5 cases)
3. Onset after initiating formula or fortifiers (6 cases; 2 overlapping with category 2)
4. Associated with hemodynamic changes (7 cases; 3 overlapping with category 1, 1 overlapping with category 2)
5. No clear associated factor (4 cases)

Median values are also shown in Table 1 to provide descriptive context; however, given the small group sizes (*n* = 3–5 per category), these values should be interpreted with caution and no statistical comparisons were performed.

1. NEC Onset Within 7 Days of Birth (5 Cases)

Case No.1 was born in poor condition due to non-reassuring fetal status, developed intraventricular hemorrhage on day 1, and NEC on day 3. Case No.2 involved severe neonatal infection and intraventricular hemorrhage on day 3, followed by NEC on day 4. Three cases (case No. 3,4,5) developed NEC on days 4, 6, and 7, respectively, with symptomatic patent ductus arteriosus (PDA) suspected. Symptomatic PDA is defined as presence of clinical signs such as hyperdynamic precordium, widened pulse pressure, tachycardia, or tachypnea, along with echocardiographic evidence of ductal significance—defined as at least one of the following: ductal diameter ≥1.5 mm, left atrium-to-aortic root ratio ≥1.5, diastolic flow velocity in the left pulmonary artery ≥0.2 m/sec, or absence of diastolic flow in the anterior cerebral artery—and requiring at least one course of indomethacin treatment. Several early-onset cases were associated with severe congenital infection, non-reassuring fetal status, or symptomatic PDA. Notably, three early NEC cases occurred at the same institution, suggesting possible differences in PDA management protocols.

2. NEC onset after approximately 2 months (5 cases)

One term infant (37 weeks, 1,300 g; case No.7) with congenital heart disease developed NEC due to hemodynamic abnormalities. Two cases (case No.6 and 8) were born at 23 weeks of gestation and were suspected to develop NEC following ophthalmologic exams. Systemic absorption of mydriatic agents may have contributed to circulatory instability. Two others (case No.9 and 10) developed NEC at 131 and 150 days of age after inguinal hernia incarceration. Both had transitioned from DHM to formula on days 15 (No.9) and 18 (No.10).

3. NEC after formula or fortifier introduction (6 cases)

Two cases (case No.9 and 10) overlapped with category 2 and were likely unrelated to formula. Case No. 11 and 12 began formula on days 12 and 13 and developed NEC on days 29 and 38, respectively, without other known causes. Case No.13 and 14 started cow's milk-based fortifier and developed NEC on days 14 and 32, respectively, with no other identified risk factors. In Japan, only cow's milk-based fortifier is available.

4. Associated with hemodynamic changes (7 cases, 4 overlapping)

Includes five cases with symptomatic PDA (NEC onset on days 4, 6, 7, 10, and 10), one case with hypoplastic left heart syndrome (case No.7; overlapping with category 2), and one with complete AV block (case No.17). In cases of symptomatic PDA and congenital heart disease, increased pulmonary blood flow and reduced systemic circulation were presumed to have led to decreased intestinal perfusion, which was considered a contributing factor to NEC onset.

5. No clear associated factor (4 cases)

These cases (case No. 18–21) had no identifiable risk factors. Although they were exclusively fed human milk, they developed to NEC between 20 and 32 days of age. All of them were extremely immature (23 and 24 weeks of gestation).

Taken together, these findings reaffirm the central role of developmental immaturity in NEC susceptibility.

## Discussion

NEC remains a major challenge in neonatal care due to its high mortality. DHM has been reported to reduce NEC incidence by approximately 50% compared to formula (risk ratio 0.53, 95% CI 0.37–0.76) (11), making it an essential preventive measure. Our observed NEC incidence of 1.58% (21/1,324 VLBW infants) was essentially identical to the rate of 1.6% recently reported in a nationwide cohort of Japanese VLBW infants (12). This similarity suggests that the NEC incidence in our DHM-based registry is consistent with national and international observations, further supporting the descriptive nature of our findings rather than indicating a differential risk attributable to DHM use.

This study aimed to identify factors contributing to NEC onset despite DHM use. Known NEC risk factors include poor birth conditions, mechanical ventilation, asphyxia, hypotension, hypothermia, fetal growth restriction, PDA, congenital heart disease, and formula feeding. This study identified cases where congenital infection, PDA, heart disease, and formula use on the first few weeks were likely contributors. Ophthalmologic exams and human milk fortifier use were observed among the cases and may warrant further investigation as possible contributing factors.

While median NEC onset is around 10 days (range: 7–23.5 days) (13), early- and late-onset cases suggest distinct etiologies. Early-onset NEC was linked to severe infection, non-reassuring fetal status and symptomatic PDA—especially in one facility, indicating a need to review PDA management there. Late-onset NEC followed hernia incarceration or ophthalmologic procedures. Systemic effects of mydriatic agents used during exams warrant further investigation.

Although not statistically significant, these cases raise a hypothesis that the timing of formula introduction may influence NEC risk; however, further studies are needed as the broader literature reports mixed findings. Exclusive human milk-based diets (EHMD), including human milk-derived fortifiers, have shown lower NEC rates than cow's milk-based alternatives (14, 15).

In the hemodynamic group, the initiation of enteral feeding tended to be delayed, possibly reflecting clinical decisions to postpone feeding until circulatory stability was achieved. In the unknown group, the main common feature was extreme prematurity and very low birth weight, despite early enteral feeding and colostrum administration. Thus, developmental immaturity may represent the primary underlying risk factor in these cases.

Although DHM and MOM are widely considered protective against NEC, they do not guarantee absolute protection; anecdotal and cohort data show that NEC can still occur despite exclusive human milk feeding. One possible explanation is interindividual variation in milk bioactive components. In particular, DSLNT—a specific human milk oligosaccharide—has been shown to reduce NEC-like injury in neonatal rat models (16), and its concentration was significantly lower in MOM samples fed to preterm infants who later developed NEC (17). These findings underscore the role of milk compositional variability in NEC risk and further support the notion that protective associations do not imply direct causation.

Because the registry scope is limited to DHM recipients, direct comparisons with non-DHM or exclusively MOM-fed infants were not feasible. To avoid implying causation, we interpret our findings descriptively and contextualize the observed NEC incidence against national cohorts and prior studies. Future work will prioritize linkage with external datasets (e.g., national neonatal registries) to enable comparative analyses across DHM, MOM, and non-DHM groups, including subgroup analyses among severe cases (Bell IIb–III).

## Limitations

This study has several limitations. First, due to the retrospective design and small sample size (*n* = 21), we did not perform statistical comparisons or multivariable modeling. For patient confidentiality, GA and BW were rounded in the individual case listings, whereas subgroup medians and IQRs were derived from the original data. This approach ensures anonymity while preserving accuracy in the descriptive statistics. Although limited modeling may be possible even with small datasets, we opted for a purely descriptive approach to avoid overinterpretation. Accordingly, our findings should be interpreted as exploratory observations rather than definitive associations.

Second, NEC case classification was based on temporal context and clinical features, which often overlapped. These categorizations were intended to identify patterns, not to imply causation. Terms such as “associated factors” has been used throughout the manuscript. Notably, developmental risk factors such as extreme prematurity and low birth weight were present in all cases and remain the most consistent predictors of NEC.

Third, several important variables were not captured in the database, including detailed DHM dosage and duration, volume of mother's own milk, specific timing of feeding advancement, and use of oropharyngeal colostrum or probiotics. However, most Japanese NICUs routinely implement probiotics and oropharyngeal colostrum administration, and feeding advancement speed has not been shown to significantly affect NEC or mortality risk in previous studies (18).

Fourth, the assessment of NEC-associated clinical factors relied on documentation and attending physicians' judgment, which may introduce some subjectivity. We aim to incorporate surgical and pathological data in future analyses to improve diagnostic accuracy. Additionally, two cases involving inguinal hernia incarceration were excluded from the final analysis, as their pathophysiology differed from classical NEC.

Fifth, our registry does not include non-DHM infants; thus, between-group comparisons could not be performed.

Despite these limitations, the study offers valuable insights into the clinical context and diversity of NEC onset among DHM recipients, and may help generate hypotheses for future prospective research.

## Conclusion

Extremely low gestational age and birth weight were common to all NEC cases in this study, underscoring their role as the most consistent and well-established risk factors for NEC. These developmental vulnerabilities likely contributed to the intestinal immaturity and impaired perfusion that predispose preterm infants to NEC, regardless of feeding practices.

While this study was descriptive in nature and limited by a small sample size, several recurring clinical features suggest that early-onset severe infections, hemodynamic instability related to PDA, and the timing of formula or fortifier introduction may play a role in NEC development, even among infants receiving DHM.

Further prospective studies are needed to determine whether delaying formula introduction, using exclusive human milk-based diets when fortification is required, and optimizing circulatory management could contribute to reducing NEC incidence in this vulnerable population.

## Data Availability

The original contributions presented in the study are included in the article/Supplementary Material, further inquiries can be directed to the corresponding author.
